# Novel Genetic Variants for Cartilage Thickness and Hip Osteoarthritis

**DOI:** 10.1371/journal.pgen.1006260

**Published:** 2016-10-04

**Authors:** Martha C. Castaño-Betancourt, Dan S. Evans, Yolande F. M. Ramos, Cindy G. Boer, Sarah Metrustry, Youfang Liu, Wouter den Hollander, Jeroen van Rooij, Virginia B. Kraus, Michelle S. Yau, Braxton D. Mitchell, Kenneth Muir, Albert Hofman, Michael Doherty, Sally Doherty, Weiya Zhang, Robert Kraaij, Fernando Rivadeneira, Elizabeth Barrett-Connor, Rose A. Maciewicz, Nigel Arden, Rob G. H. H. Nelissen, Margreet Kloppenburg, Joanne M. Jordan, Michael C. Nevitt, Eline P. Slagboom, Deborah J. Hart, Floris Lafeber, Unnur Styrkarsdottir, Eleftheria Zeggini, Evangelos Evangelou, Tim D. Spector, Andre G. Uitterlinden, Nancy E. Lane, Ingrid Meulenbelt, Ana M. Valdes, Joyce B. J. van Meurs

**Affiliations:** 1 Department of Internal Medicine, Erasmus Medical Center, Rotterdam, The Netherlands; 2 California Pacific Medical Center Research Institute, San Francisco, California, United States of America; 3 Department of Medical Statistics and Bioinformatics, Section Molecular Epidemiology. Leiden University Medical Center, Leiden, The Netherlands; 4 Department of Twins Research and Genetic Epidemiology Unit, King’s College London, London, United Kingdom; 5 Thurston Arthritis Research Center, University of North Carolina, Chapel Hill, North Carolina, United States of America; 6 Duke Molecular Physiology Institute and Division of Rheumatology. Duke University School of Medicine, Durham, North Carolina, United States of America; 7 Departments of Medicine and Epidemiology & Public Health, University of Maryland School of Medicine, Baltimore, Maryland, United States of America; 8 Geriatrics Research and Education Clinical Center, Baltimore Veterans Administration Medical Center, Baltimore, Maryland, United States of America; 9 Health Sciences Research Institute, University of Warwick, Warwick, United Kingdom; 10 Department of Epidemiology, Erasmus Medical Center, Rotterdam, The Netherlands; 11 Department of Epidemiology, Harvard T.H. School of Public Health, Boston, Massachusetts, United States of America; 12 School of Medicine, University of Nottingham, Nottingham, United Kingdom; 13 Epidemiology Division, Family Medicine and Public Health Department, University of California, San Diego, La Jolla, California; 14 Respiratory, Inflammation, Autoimmunity Innovative Medicines, AstraZeneca AB, Mölndal, Sweden; 15 Nuffield Department of Orthopaedics, Rheumatology and musculoskeletal sciences, University of Oxford, United Kingdom; 16 Department of Orthopaedics, Leiden University Medical Center, Leiden The Netherlands; 17 Department of Rheumatology and Department of Clinical Epidemiology, Leiden University Medical Center, Leiden, The Netherlands; 18 University of California at San Francisco, San Francisco, California; 19 University Medical Center Utrecht, Utrecht, The Netherlands; 20 Decode Genetics, Reykjavik, Iceland; 21 Wellcome Trust Sanger Institute, Hinxton, United Kingdom; 22 Department of Hygiene & Epidemiology, University of Ioannina School of Medicine, Ioannina, Greece; 23 Department of Epidemiology and Biostatistics, School of Public Health, Imperial College London, London, United Kingdom; 24 School of Medicine, University of California, Davis, Sacramento, California; Stanford University School of Medicine, UNITED STATES

## Abstract

Osteoarthritis is one of the most frequent and disabling diseases of the elderly. Only few genetic variants have been identified for osteoarthritis, which is partly due to large phenotype heterogeneity. To reduce heterogeneity, we here examined cartilage thickness, one of the structural components of joint health. We conducted a genome-wide association study of minimal joint space width (mJSW), a proxy for cartilage thickness, in a discovery set of 13,013 participants from five different cohorts and replication in 8,227 individuals from seven independent cohorts. We identified five genome-wide significant (GWS, P≤5·0×10^−8^) SNPs annotated to four distinct loci. In addition, we found two additional loci that were significantly replicated, but results of combined meta-analysis fell just below the genome wide significance threshold. The four novel associated genetic loci were located in/near *TGFA* (rs2862851), *PIK3R1* (rs10471753), *SLBP/FGFR3* (rs2236995), and *TREH/DDX6* (rs496547), while the other two (*DOT1L* and *SUPT3H/RUNX2*) were previously identified. A systematic prioritization for underlying causal genes was performed using diverse lines of evidence. Exome sequencing data (n = 2,050 individuals) indicated that there were no rare exonic variants that could explain the identified associations. In addition, *TGFA*, *FGFR3* and *PIK3R1* were differentially expressed in OA cartilage lesions versus non-lesioned cartilage in the same individuals. In conclusion, we identified four novel loci (*TGFA*, *PIK3R1*, *FGFR3 and TREH*) and confirmed two loci known to be associated with cartilage thickness.The identified associations were not caused by rare exonic variants. This is the first report linking *TGFA* to human OA, which may serve as a new target for future therapies.

## Author Summary

Osteoarthritis (OA) is the most common form of arthritis and a leading cause of chronic disability in the western society affecting millions of people. OA is a degenerative joint disease characterized by changes in all joint tissues, including cartilage, bone and synovium, causing chronic pain and loss of function. There are no effective therapeutic treatments available for OA and therefore finding novel biological pathways through genetic association studies can open up new treatment options. The number of known DNA variants associated with OA-risk is limited.

To identify new loci, we have performed a Genome Wide Association Study meta-analysis on cartilage thickness, one of the joint tissues affected in OA in a total sample of more than 20,000 individuals from twelve cohorts. This analysis revealed six variants associated with cartilage thickness, four of these being novel associations, including TGFA as the most prominent one. A systematic prioritization for underlying causal genes, using diverse lines of evidence, highlighted genes underlying the disease associations, including TGFA, RUNX2 and PIK3R1. Large scale exome sequencing data (n = 2,050 individuals) indicated that there were no rare exonic variants that could explain the identified associations. This is the first report linking *TGFA* to human OA, which may serve as a new target for future therapies

## Introduction

In spite of advances in the understanding of OA, the absence of effective therapeutic targets demonstrates that a better comprehension of its causes and pathophysiological mechanisms is needed. Since genome-wide genetic studies are hypothesis-free and do not suffer from the bias of previous knowledge, they have the potential to identify novel biological pathways involved in OA. The discovery of novel genes has the potential to identify novel treatment options. In addition, more personalized medicine approaches for OA can be explored through prediction of risk for disease as well as classification of disease subtypes.

Heritability of hip OA has been estimated to be around 40–60%. However, to date only few genetic variants have been successfully identified [1,2]. The reasons for finding only a modest number of genetic loci associated with hip OA can be attributed partially to relatively modest samples sizes in comparison to other complex diseases, such as myocardial infarction [3]. In addition, phenotype heterogeneity is an important issue in OA genetics as this is well known to reduce power to robustly detect signals. The problem of heterogeneity in genetic association studies of OA has been highlighted before [4,5] and is exemplified by the fact that the definition of the phenotype is a combination of bone and/or cartilage features as well as clinical complaints. Moreover, there is growing consensus that OA can be divided into multiple sub-phenotypes each with their own etiology and risk factors. For example, it has been demonstrated that individuals with hip OA, where only cartilage degradation is involved (atrophic OA form), are linked to a different systemic bone phenotype compared to individuals with OA where bone formation is also present [6].

As a way to overcome this, we examined a quantitative trait, which is one of the structural components of joint health, cartilage thickness, as a distinct phenotype.

Joint Space Width (JSW) is considered to be a proxy for cartilage thickness measured on hip radiographs. Minimal JSW (mJSW) has been shown to be a more reliable measure for hip joint health compared to the classical Kellgren & Lawrence score [7]. Previously, we have demonstrated that using only a modest discovery sample size (n = 6,000), we were able to successfully identify a genome-wide significant association of the *DOT1L* locus with mJSW as well as hip OA [1,8]. We now aimed to perform a more powerful analysis by combining data from five studies in the discovery phase, and subsequent replication in seven additional studies, amounting to a total sample size of 21,240 to identify new genes implicated in joint health using mJSW as a proxy for cartilage thickness. Using whole exome sequence data from 2,050 individuals we screened the discovered genes for potential functional variants. Subsequently we used multiple approaches that leverage different levels of information to enforce evidence of candidate genes annotated close to the associated signals.

## Results

### Identification of novel genetic loci associated with cartilage thickness

Genome-wide Association analysis of mJSW of the hip with genetic variants was performed in a discovery set that included 13,013 individuals (see S1 Table and S1 Text for cohort specifics) with data on ±2,5 million genotyped or HapMap Phase II imputed SNPs. We applied extensive quality control measures (see S2 Table and S3 Table for details on quality control and exclusion criteria) leaving a total of 2,385,183 SNPs available for association analyses. Genomic control inflation factors for the P values of the RS, TwinsUK, MrOS, and SOF GWAS were low (λ = 1.02, 1.01, 1.02 and 0.99 respectively), and the interquantile-quantile plot (S1 Fig) also indicated no residual population stratification due to cryptic relatedness, population substructure or other biases.

**Fig 1:**
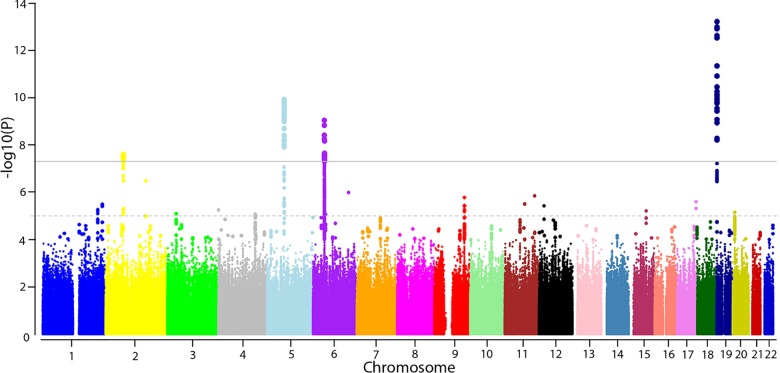
Manhattan plot for association of mJSW in the discovery phase. The -log10 P values, for each of the 2.5 million tests performed as part of the genome wide association of minimal joint space width of the hip (mJSW), plotted against their position per chromosome. Full results of the discovery GWAS are accessible through www.glimdna.org. The gray solid horizontal line corresponds to the genome-wide significant threshold (P = 5x10-8). The dotted grey line corresponds to the selection for replication threshold (P = 1x10^-5^).

The discovery analysis yielded eighteen independent SNPs with suggestive evidence for association (P <1*10^−5^) with mJSW, of which five (four genetic loci) met the genome-wide significance threshold of P-value ≤ 5*10^−8^ (see Fig 1). The top SNPs from these eighteen loci were selected for replication in additional 8,227 individuals from seven different cohorts. We observed that six of the eighteen SNPs significantly replicated (P<0.05) with the same direction of effect (see Table 1). When we combined discovery and replication results in a meta-analysis, the five SNPs that met genome-wide significance in the discovery analysis became more significant and another two SNPs that replicated in independent cohorts reached suggestive evidence (P≤ 1*10^−6^) for association in the combined meta-analysis.

**Table 1:** Results from the minimal Joint Space Width genome-wide association study; discovery, replication and joint meta-analysis

*SNP*	*Chr*	*Position*	*Allele 1*	*Allele 2*	*Freq 1*	*Discovery*			*Replication*			*Joint meta-analysis*			*Locus*
						*Beta*	*SE*	*P-value*	*Beta*	*SE*	*P-value*	*Beta*	*SE*	*P-value*	
rs11880992	19	2127403	A	G	0,40	0,096	0,013	9,1E-14	0,091	0,029	1,5E-03	0,095	0,012	3,2E-16	DOT1L
rs2862851	2	70566310	T	C	0,49	-0,070	0,013	2,9E-08	-0,062	0,018	4,8E-04	-0,067	0,010	5,2E-11	TGFA
rs10948155	6	44795935	T	C	0,67	0,081	0,013	1,2E-09	0,045	0,019	1,5E-02	0,069	0,011	1,5E-10	SUPT3H-RUNX2
rs12206662	6	45484199	A	G	0,07	-0,187	0,032	7,9E-09	-0,093	0,035	7,3E-03	-0,140	0,024	1,3E-09	SUPT3H/RUNX2
rs10471753	5	67854708	c	G	0,61	0,082	0,013	1,5E-10	0,019	0,019	3,3E-01	0,062	0,011	3,8E-09	PIK3R1
rs496547	11	118081673	A	T	0,34	-0,064	0,013	1,5E-06	-0,046	0,022	3,8E-02	-0,058	0,011	1,5E-07	TREH-DDX6
rs2236995	4	1674340	T	G	0,49	0,057	0,013	6,4E-06	0,038	0,018	3,2E-02	0,049	0,010	9,7E-07	SLBP
rs717433	20	7786135	T	C	0,76	0,067	0,015	8,0E-06	0,058	0,033	8,3E-02	0,065	0,014	1,4E-06	HAO1
rs10495106	1	217017809	T	C	0,94	0,119	0,026	6,2E-06	0,055	0,037	1,3E-01	0,096	0,021	6,4E-06	TGFB2
rs6592847	11	78889654	A	G	0,28	0,070	0,015	3,7E-06	-0,014	0,056	8,1E-01	0,065	0,015	9,5E-06	NARS2
rs6429001	1	235495143	A	G	0,83	0,078	0,017	3,1E-06	-0,006	0,039	8,8E-01	0,065	0,015	2,4E-05	RIR2
rs13148031	4	145679788	A	G	0,60	0,057	0,013	9,8E-06	0,016	0,018	3,6E-01	0,043	0,010	3,5E-05	HHIP-GYPA
rs2061109	3	35380584	C	G	0,50	0,057	0,013	8,8E-06	0,003	0,030	9,2E-01	0,048	0,012	3,7E-05	ARPP-21
rs6437120	2	158493396	T	C	0,50	-0,064	0,013	4,0E-07	0,006	0,019	7,5E-01	-0,043	0,011	4,5E-05	ACVR1-UPP2
rs4837613	9	118349346	c	G	0,49	-0,062	0,013	2,0E-06	0,002	0,019	9,1E-01	-0,041	0,011	1,1E-04	ASTN2
rs11045356	12	20683368	A	G	0,17	0,078	0,017	4,4E-06	-0,052	0,036	1,5E-01	0,059	0,016	1,8E-04	PPE3A
rs2703529	17	74709369	A	G	0,07	-0,114	0,024	2,9E-06	0,006	0,034	8,6E-01	-0,073	0,020	1,9E-04	HRN3P3
rs7739938	6	139823110	A	G	0,03	-0,314	0,065	1,1E-06	-0,030	0,026	2,5E-01	-0,069	0,024	3,9E-03	CITED2

Chr: Chromosome; Allele 1: Modelled allele in the analysis; Allele 2: alternative allele; SE: standard error.

The top signal in the combined meta-analysis, rs1180992 (Table 1, P_combined_ = 3.2x10^-16^), is located in the intronic region of the previously OA associated *DOT1L* gene. This variant is very close to and in linkage disequilibrium with rs12982744 (D’ = 1, r^2^ = 1), which was previously found in association with mJSW and hip OA [1,8].

**Fig 2:**
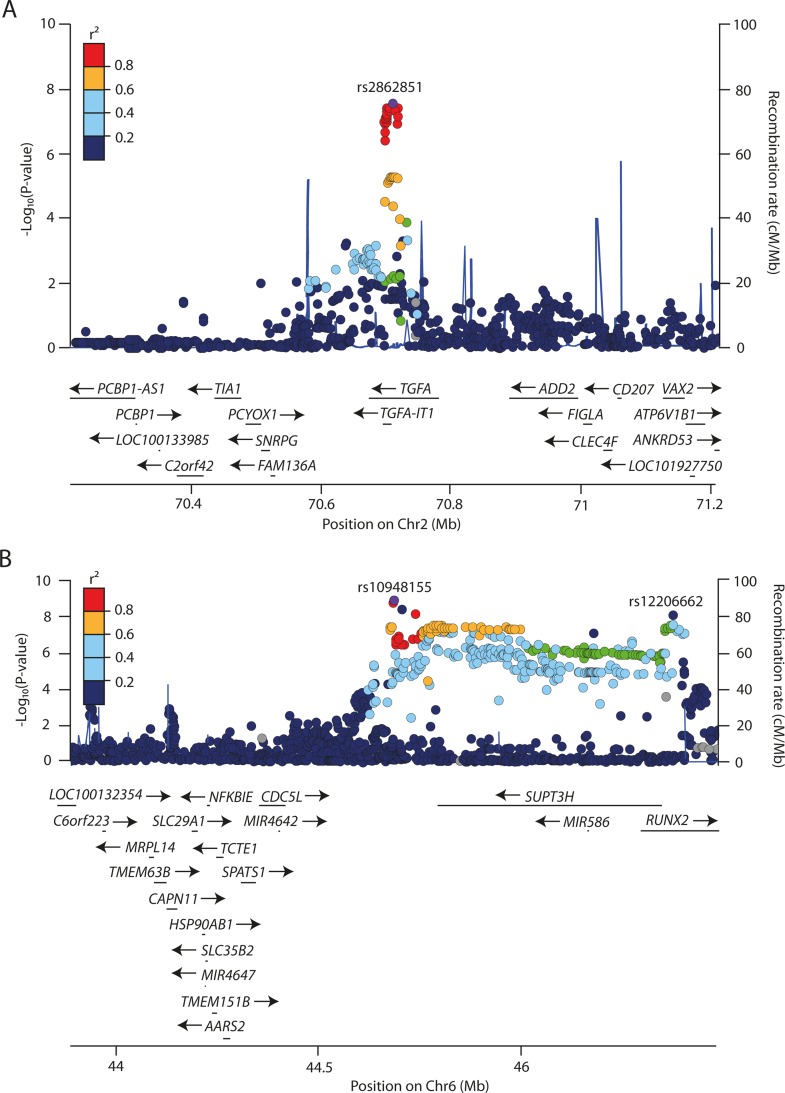
Regional association plot for the locus of rs2862851 rs12206662/rs10948155. SNPs are plotted by position in a 800-kb window versus association with mJSW (−log10 P) for A. rs2862851 (TGFA locus) and B. rs12206662/rs10948155 (SUPT3H/RUNX2 locus). The purple dot highlights the most significant SNP in discovery analysis. Blue peaks indicate recombination rates. The SNPs surrounding the most significant SNP are color coded to reflect their LD with this SNP (from pairwise r^2^ values from the HapMap CEU). Genes, exons and the direction of transcription from the University of California at Santa Cruz genome browser are depicted. Plots were generated using Locus Zoom [9]

The *DOT1L* signal was followed in strength of association by rs2862851 (P_combined_ = 5.2x10^−11^), which is annotated to the intronic region of *TGFA* (Fig 2A). Two variants near *RUNX2*, rs10948155 and rs12206662, also reached genome-wide significance for association with mJSW (Fig 2B). The two variants in the RUNX2 locus were weakly correlated (r^2^<0.2). Conditional analysis, using GCTA, showed that both SNPs represented different signals (S4 Table). Finally, the last signal that reached genome-wide significance was rs1047175*3*, an intergenic variant closer to *PIK3R1* (~450 Kb) than to *SLC30A5* (~750Kb) (Table 1, P_combined_ = 3.8*10^−9^).

Other suggestive signals for association with mJSW at a P_combined_≤ 1x10^−6^ including signals with significant replication were rs496547 (p = 1.5x10^−7^), a downstream gene variant located 3' of *TREH* and, an intron variant annotated near *SLBP* (rs2236995; p = 9x10^−7^). All other additional signals selected in the discovery stage did not replicate.

### Association of identified loci with hip OA and other musculoskeletal phenotypes

We examined whether the five GWS and two suggestive mJSW loci were also associated with hip OA in a total of 8,649 cases and >57,000 controls. Detailed description of the cohorts and OA definitions is given in S1 Table. Table 2 shows the associations found with hip OA. We observed that five of the seven identified mJSW loci were also associated with hip OA (p-value<0.05). Apart from the known *DOT1L* locus, the variant near *TGFA* was significantly associated with hip OA (Table 2, P = 4.3x10^−5^). In addition, the SNP near *SLBP* and the two SNPs near *RUNX2* were associated with hip OA. One of the latter SNPs, rs10948155, is in high LD with a variant (rs10948172, D’ = 0.95 and r^2^ = 0.90) previously found in association with hip OA in males at borderline GWS level (2). However, in our study, rs10948155 was just marginally associated with hip OA in the overall analysis (Table 2, P = 0.021). We observed the second variant in this genomic region, an intronic variant in *RUNX2*, rs12206662, to have a larger effect size (β = 0.14, P = 1.1×10^−4^ r^2^ = 0.09 with rs10948172).

**Table 2:** Association of mJSW loci with Hip OA

*SNP*	*Locus*	*Modelled allele*	*Beta*	*SE*	*P-value*
rs11880992	DOT1L	A	-0,064	0,016	6,4E-05
rs2862851	TGFA	T	0,059	0,016	4,3E-05
rs10948155	SUPT3H-RUNX2	T	-0,037	0,016	2,1E-02
rs12206662	SUPT3H-RUNX2	A	0,140	0,036	1,1E-04
rs10471753	PIK3R1	C	0,006	0,016	6,9E-01
rs496547	TREH-DOX6	A	0,006	0,016	7,1E-01
rs2236995	SLBP	T	-0,046	0,015	1,7E-03

SE: standard error

We further examined whether the identified loci were found associated with other phenotypes in earlier reports (Table 3). Five of the seven identified mJSW SNPs mapped to loci that have previously been associated with other bone-related phenotypes, primarily height. However, many of the identified height loci were not highly correlated with the mJSW signal (Table 3). Additional adjustment for height did not have an effect on the described association with mJSW; they showed an independent, possibly pleiotropic effect, on both traits. A particularly dense number of associations with different bone related phenotypes were present in the *RUNX2* 5’ region, where variants have been associated to BMD [10], height [11], osteoarthritis [2] and ossification of the spine [12]. Given the low LD between the variants underlying the different GWAS signals, it is likely that these represent independent associations.

**Table 3:** Association of mJSW loci with previously reported phenotypes

*SNP*	*Locus*	*Phenotype*	*Reported SNP*	*r^2^ with mJSW SNP*
**rs2862851**	TGFA	-	-	
**rs11880992**	DOT1L	OA[1]	*rs12982744*	*1*
** **		Height[13]	*rs2523178*	*0*,*1*
**rs12206662**	SUPT3H-RUNX2	OA[2]	*rs10948172*	*0*,*09*
** **		Height[13]	*rs10948222*	*0*
** **		BMD[10]	*rs117755164*	*0*,*04*
** **		OPLL[12]	*rs927485*	*0*
**rs10948155**	SUPT3H-RUNX2	OA[2]	*rs10948172*	*0*,*84*
		Height[13]	*rs10948222*	*0*
		BMD[10]	*rs117755164*	*0*,*21*
		OPLL[12]	*rs92748*	*0*
**rs10471753**	PIK3R1	-	* -*	
**rs2236995**	SLBP	Height[14]	*rs2247341*	*0*,*6*
**rs496547**	TREH-DDX6	Height[14]	*rs494459*	*0*,*36*
		Metabolite levels[15]	*rs507080*	*0*,*21*
		Vitiligo[16]	*rs638893*	*0*,*02*
		Celiac disease/ RA[17]	*rs10892279*	*0*,*36*
		SLE[18]	*rs4639966*	*0*,*21*

### Prioritization of genes underlying the genetic loci

We used multiple approaches that leverage different levels of information (e.g., gene expression, regulatory regions, published literature, mouse phenotypes) to prioritize candidate genes at each mJSW locus. Table 4 shows the summarized results from these analyses. In addition to the seven loci identified in the current study, we also analyzed five previously published loci for hip OA [2].

**Table 4:** Prioritized genes for Osteoarthritis loci. Biological and database evidence was collected to identify the causal gene in each investigated osteoarthritis associated locus. Locus gene sets were constructed by taking a region of 500 Kb upstream and 500Kb downstream of the lead SNP of that locus (1MB locus region), in total we analyzed 152 genes. The table summarizes the biological evidence for each osteoarthritis associated locus: 1) Nearest located genes; 2) DEPICT gene prioritization results, only genes that were significantly prioritized (FDR <0.05) are listed; 3) GRAIL results, only nominal significant results are listed (P<0.05); 4) Mouse cartilage or bone gene expression; 5) Bone or cartilage development phenotype in mouse. Mouse expression and phenotype data were obtained from the Jackson lab database (http://www.informatics.jax.org/); 6) Human cartilage tissue gene expression; 7) differential gene expression between OA-lesioned cartilage tissue and non-lesioned cartilage tissue [19]; 8) OMIM phenotype; 9) eQTL evidence [20]; 10) Nonsynonymous variants in LD >0.6 with lead SNP. All evidence for each gene is summarized, genes with the highest number of different types of evidence are reported.

	Nearest Genes	PIK3R1	KLHL42 PTHLH	CDC5L; SUPT3H	GNL3	DOT1L	RUNX2	SLBP	TGFA	TRH; DDX6	ZMYND8; NCOA3	IGFBP3	CHST11	FILIP1; SENP6
**GRAIL results**	**Gene**		PTHLH								SULF2	IGFBP3		
	**GRAIL P-value**		6,46E-03								2,40E-02	4,37E-03		
**DEPICT Results**	**Gene**	PIK3R1	KLHL42	RUNX2			RUNX2		VAX2	UPK2		IGFBP3	CHST11	
	**DEPICT P-value**	7,35E-05	5,03E-03	1,51E-03			1,51E-03		8,85E-04	3,81E-03		2,37E-03	1,04E-03	
**NS variants**	**nr. variants**	-	-	-	8	1	-	-	-	-	1	-	-	-
	**Gene**	-	-	-	GNL3;NEK4; STAB1; SPCS1; ITIH1	DOT1L	-	-	-	-	NCOA3	-	-	-
**eQTL**	**Gene**	-	-	SUPT3H	NT5DC2	AP3D1	**-**	SLBP	**-**	**-**	**-**	**-**	**-**	**-**
	**cis-eQTL P-value**	-	-	4,16E-22	1,07E-10	1,10E-15	**-**	6,75E-10	**-**	**-**	-	-	**-**	**-**
	**eQTL tissue**	-	-	Blood	blood	Blood	**-**	Blood	**-**	**-**	**-**	**-**	**-**	**-**
**OMIM**	**Gene**	PIK3R1	PTHLH	RUNX2	-	-	RUNX2	FGFR3	**-**	KMT2A	-	-	-	-
	**OMIM Disorder**	SHORT syndrome	Brachy- dactyly	Cleido—cranial dysplasia	-	-	Cleido—cranial dysplasia	skeletal dysplasia	-	Wiedemann-Steiner syndrome	-	-	-	-
Gene Expression Mouse*	**Gene**	-	PTHLH	RUNX2	Multiple genes	GADD45B	RUNX2	FGFR3	TGFa	KMT2A	SULF2	IGFBP3	CHST11	COL12A1
	**B&C expression**	-	+	+	+	+	+	+	+	-	+	+	+	+
	**B&C Phenotype**	-	+	+	+	-	+	+	-	+	-	+	+	+
Gene Expression Human**	**Gene**	PIK3R1	PTHLH	RUNX2	GNL3	GADD45B	RUNX2	FGFR3/SLBP	TGFa	-	SULF2/NCOA3	IGFBP3	-	COL12A1
	**Cartilage tissue**	+	+	+	+	+	+	+	+	-	+	+	-	+
	Diff. expression in OA*	PIK3R1	PTHLH	-	GNL3	GADD45B	-	FGFR3/SLBP	TGFa	Multiple genes	NCOA3	IGFBP3	TXNRD1	COL12A1
**Prioritized Gene**	**Prioritized Gene**	**PIK3R1**	**PTHLH**	**RUNX2**	**GNL3**	**DOT1L**	**RUNX2**	**FGFR3**	**TGFa**	**KMT2A**	**SULF2**	**IGFBP3**	**CHST11**	**COL12A1**
	**Lines of Evidence**	**5**	**6**	**5**	**4**	**3**	**6**	**5**	**4**	**2**	**4**	**7**	**4**	**4**
	**Alternate Gene**	-	-	-	-	*GADD45B*	-	*SLBP*	-	*UPK2*	*NCOA3*			
	**Lines of Evidence**					*3*		*5*		*2*	*4*			

* If multiple genes were expressed in cartilage, the gene with also a skeletal phenotype (in KO) is shown. However, the lines of evidence are counted for each gene.

** For some of the loci, multiple genes in the 1MB region surrounding the identified SNP were found to be significantly expressed. In the table the gene(s) are indicated that have additional lines of evidence

First, we focused on two gene prioritization methods: (i) DEPICT, a novel tool designed to identify the most likely causal gene in a given locus, and identify gene sets that are enriched in the genetic associations [21], and (ii) GRAIL which uses existing literature to identify connections between genes in the associated loci [22]. The DEPICT analysis yielded seventeen significantly prioritized genes (FDR >0.05), from which three genes were also significantly prioritized in the GRAIL analysis (S5 Table and S6 Table). Next, using the Online Mendelian Inheritance in Man (OMIM) database (http://omim.org), we identified genes with mutations implicated in abnormal skeletal growth in humans; for 50% of the loci, a skeletal syndrome gene was present (S7 Table). Similarly, we investigated if any of the genes had a known bone and cartilage development phenotype in mice. Very similar to the human phenotypes, the mice knockouts of the same genes resulted in bone and cartilage phenotypes (http://mousemutant.jax.org/) (S7 Table). Other supporting biological evidence that we gathered consisted of known expression quantitative loci (eQTL) and nonsynonymous variants in LD (r^2^>0.6) with the lead SNP of a locus (S8 Table and S9 Table), as well as expression in bone and cartilage tissue in mice using data from the Jackson lab database (S7 Table).

To further explore which genes are possibly underlying the genetic associations identified in this study, we analyzed gene expression in a paired set of non-lesioned and OA-lesioned cartilage samples of the RAAK study acquired from 33 donors at the time of joint replacement surgery for primary OA [19]. We first examined which genes are expressed in a set of seven human healthy cartilage samples (S7 Table). Additionally, we tested which of the genes located in 1MB region surrounding the lead SNP were differentially expressed in OA-lesioned cartilage versus non-lesioned cartilage of the same hip. Of the 152 genes that were selected, 129 genes were represented on the array. Of those, 64 genes were significantly expressed in the cartilage samples. For eight of the twelve loci, we found genes that were differentially expressed in OA lesioned cartilage versus non-lesioned cartilage (Table 4, S10 Table). Differential expression in cartilage healthy vs OA affected cartilage was performed likewise (S10 Table), while additionally adjusting for sex and age. Given the relatively small number of healthy samples (n = 7) with large age range these data are less robust and we did not use these data in gene prioritization.

For each gene a prioritization score was computed, based on equally weighting of the ten lines of evidence (Table 4). Following this approach, *RUNX2* is highly likely to be the causal gene associated with *rs12206662* and rs10948155. Similar strong evidence is found for *rs788748 (IGFBP3)* and *rs10492367* (*PTHLH)*. In addition, suggestive evidence for a causal gene is found for the following: rs10471753 (*PIK3R1*), rs835487 (*CHST11*), rs2862851 (*TGFA*), rs6094710 *(SULF2)*, rs9350591 (*COL12A1)* and rs11177 (*GNL3*). However for some loci the current evidence is ambiguous, suggesting more than one gene as the potentially causal one; *rs2236995 (FGFR3 or SLBP)*, rs11880992 (*GADD45B* or *DOT1L*) and rs496547*(KMT2A or UPK2)* (Table 4).

### Exome sequencing of prioritised genes

In 2,628 individuals from the Rotterdam Study, exome sequencing was performed at a mean depth of 55x. Of those, 2,050 individuals also had mJSW and hip OA phenotype data. Baseline characteristics of those individuals were similar to the source population, mean age was 67.3 years, 57% of the individuals were female and mean of mJSW was 3.81 mm (SD 0.82). Details of the experimental procedure and variant calling are given in the supplementary material (S2 Text). Only the variants with a minimal allele count of three in the total population were selected for analysis. Within the sixteen prioritized genes, a total number of 158 variants were identified in the protein-coding region, of which 85 were non-synonymous and one was a stop-gain mutation (Table 5, S11 Table).

**Table 5:** Association between protein coding variants identified by exome-sequencing and mJSW.

	Variants Tested		
Gene	non-synonymous	STOP Gained	mJSW* Pvalue
**CHST11**	3		**0,05**
COL12A1	23	1	1,00
DOT1L	13		1,00
FGFR3	12		0,64
GADD45B	1		0,91
GNL3	5		0,64
IGFBP3	2		0,28
KMT2A	8		0,40
NCOA3	7		0,24
PIK3R1	3		0,80
PTHLH	0		NA
**RUNX2**	2		**0,05**
SLBP	1		0,16
**SULF2**	3		**0,05**
**TGFA**	1		**0,04**
UPK2	1		0,74

*A burden test was performed to test the association between the protein-coding variants in the candidate genes and mJSW

We first performed a single variant test, where we tested each of the 86 variants changing the amino-acid sequence for association with the mJSW trait (S11 Table). We observed four nominal significant associations, with rare variants in *SULF2*, *TGFA*, *RUNX2* and *FGFR3*. None of these rare exonic variants explained the original association between the GWAS hit and mJSW or hipOA when tested in a multivariate model (S12 Table). Next, we performed a burden test (SKAT) [23], to investigate whether the cumulative effects of the variants present in the sixteen selected genes were associated to mJSW, while adjusting for age and gender (Table 5). We observed a nominal significance burden test (p<0.05) for *TGFA*, *SULF2*, *CHST11* and *RUNX2* for mJSW. However, none of these findings reached significance after correction for multiple testing.

### mJSW associated SNPs in regulatory regions

**Fig 3:**
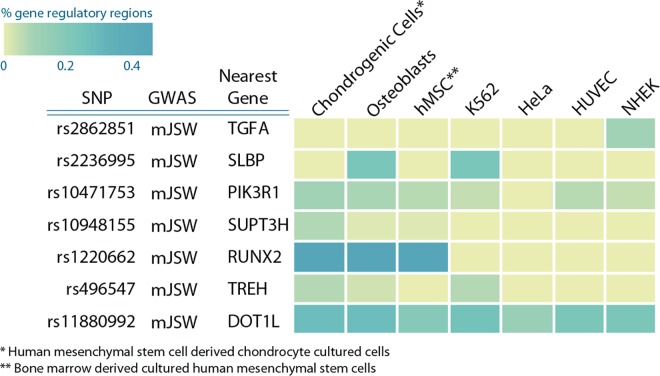
mJSW and OA associated variants are co-localized with potential gene regulatory markers. We examined the epigenetic histone marks in Chondrogenic cells, osteoblasts, hMSC, K562, HUVEC, HeLA and NHEK cells. This heatmap of the percentage of variants in gene regulatory regions (enhancer/promoter associated regions) in high LD (r^2^ >0.8) with lead GWAS SNP. Enrichment was calculated according [25].

For most of the loci, no obvious protein-coding variants were found that could explain the associations. In previous studies it was shown that disease-associated variants are enriched in regulatory DNA regions [24,25]. We therefore examined whether the identified DNA variants (or SNPs in high LD) resided in chondrocyte and/or osteoblast specific enhancer regions, using data from ENCODE and ROADMAP [26–28]. To this end, we compared CHIP-seq signals from five different chromatin state markers (H3K4me3, H3K4me1, H3K36me3, H3K27me3, H3K9me3) in chondroblasts and osteoblasts to four cell lines from another origin. Together, these chromatin state markers identify promoter and enhancer activity in each of the cell lines. With the exception of *rs2862851*, *w*e observed that for all mJSW genetic loci, SNPs in high LD were located in cell regulatory regions in chondroblast and/or osteoblast cells (see Fig 3 for an overview and S13–S18 Tables for each locus).

## Discussion

Only a modest number of genetic variants has been successfully identified through genome-wide association studies for OA This can in part be explained by the phenotypic heterogeneity of OA. Therefore, we used mJSW, a proxy for cartilage thickness in the hip joint, as one of the structural components of joint health. An additional advantage of this phenotype is its continuous nature, which increases power compared to a dichotomous trait, such as OA-status. We identified six independent loci associated with cartilage thickness in the hip joint, of which four surpassed genome-wide significance (*TGFA*, *PIK3R1*, *SUPT3H-RUNX2*, *DOT1L*) and two were suggestive for association with mJSW (*SLBP/FGFR3*, *TREH-DDX6*). Four of these loci (*TGFA*, *SUPT3H-RUNX2*, *DOT1L* and *FGFR3*) were also associated with hip OA.

The fact that we were able to identify six loci with the current sample size (13K individuals in the discovery) indicates that cartilage thickness is a phenotype providing a better yield in number of discoveries than the efforts ran with traditional composite radiographic scores. As a comparison, the largest GWAS study up to now, arcOGEN with 7,4K cases and 11K controls as discovery, yielded one locus in the overall analysis, and seven additionally in a number of stratified analyses. Interestingly, in the current manuscript we report on rs10948155, which is in high LD (r^2^ >0.8) with a locus from arcOGEN which was only marginally associated (p below genome-wide significance threshold) with OA in males only [2]. By using a cartilage specific endophenotype, evidence for this locus is elevated here to genome-wide significance in the total population, underscoring the increased power when more specific endophenotypes are used. Endophenotypes are quantifiable biological traits intermediate in the causal chain between genes and disease manifestation (in this case osteoarthritis). JSW can be precisely measured throughout the life of individuals [7] and also displays variation in normal subjects. Therefore, mJSW may be more tractable for the genetic dissection of OA.

Across the cohorts in this manuscript, mJSW has been measured in different ways, using both hand measured JSW on radiographs as well as (semi) automatic software which could have added some noise to the overall meta-analysis. Future cross-calibration of JSW measurements might aid in a more precise measurement and additional power to pick up genetic loci.

To the best of our knowledge, we are the first to scrutinize exome variants in relation to OA identified by large scale re-sequencing. We did not find low frequency exonic variants in any of the prioritized genes that could explain the observed associations with mJSW. We do have to keep in mind that the power of the exome sequencing effort is smaller than the original discovery analysis. We were unable to examine variants with allele frequencies below 0,07%. In addition, for rare or low allele frequencies, we only had power to detect relatively large effect sizes. For example, we had 80% power to detect a beta of 0,7 mm (almost 1SD) difference for a variant with 1% allele frequency. However, we tested all of the discovered exome variants in a multivariate analysis, and found that the novel identified rare exome variants did not affect the association between the GWAS-identified variants and mJSW in the same sample. This suggests that the associations between mJSW and the identified SNPs are not explained by rare exonic variants and likely exert their effects through regulation of expression. Indeed, supporting this hypothesis, we found that many these variants (or SNPs in LD) were annotated in regions that were annotated as regulatory active in chondroblastic and/or osteoblastic cells. However, more work is needed to examine the exact biological mechanism underlying the identified genetic loci.

*TGFA* (Transforming Growth Factor Alpha, *rs2862851)* was the strongest novel locus associated with cartilage thickness and hip OA. *TGFA* has been suggested to be involved in endochondral bone formation in mice, specifically the transition from hypertrophic cartilage to bone [29]. Recent, *TGFA* has also been implicated in the degeneration of articular cartilage during OA in rats [30]. Our results now imply a relationship between TGFA and human OA. In addition to the genetic association, we also show that TGFA expression is higher in human OA affected versus non-lesioned cartilage, possibly indicating that TGFA has a role in cartilage remodeling.

Functional characterization of the TGFA- associated locus by an examination of the histone methylation marks representing promoter or enhancer activity, did not reveal an obvious explanation for the functional impact of the SNP. However, the examined histone mark data represent unstimulated cells, and it is anticipated that the promoter and enhancer activity change upon stimulation of the cells. It is becoming more clear that effects of SNPs can be stimulus and context dependent, as has recently been shown for human monocytes, where many regulatory variants display functionality only after pathophysiological relevant immune stimuli [31].

The identified *SUPT3H-RUNX2* locus contains two variants, rs12206662 and rs10948155, which are partially independent of each other. Where rs12206662 is located in the first intron of the *RUNX2* gene near the second transcription start site (the so-called P2 promoter), rs10948155 is located more than 500kb away from *RUNX2* between *CDC5L* and *SUPT3H*. However, rs10948155 is in high linkage disequilibrium with SNPs near in the P2 promoter and SNPs located in chondroblast specific enhancer regions (S16 and S17 Tables).Possibly, these enhancer regions regulate *RUNX2* gene expression during endochondral differentiation. *RUNX2* (Runt-related transcription factor 2) is a master transcription factor for controlling chondrocyte hypertrophy and osteoblast differentiation [32]. Previous genome-wide association studies have identified variants in the *SUPT3H-RUNX2* locus associated with other bone and cartilage related phenotypes including height [14], bone mineral density [10] and ossification of the posterior longitudinal ligament of the spine [12]. All these previously published loci are independent of the two mJSW SNPs identified in the current study. We hypothesize that the SNPs are located in long-range enhancers, which regulate *RUNX2* gene expression during endochondral differentiation via a chromatin-loop mediating protein.

We have also identified rs10471753, with *PIK3R1* (Phosphoinositide-3-Kinase, Regulatory subunit 1 alpha) as the closest and strongest prioritized gene, related to rs10471753 associated with mJSW. Mutations in this gene are known to cause the SHORT syndrome, which is a rare multisystem disease with several manifestations including short stature, hernias, hyper extensibility and delayed dentition [33].Taken together with the fact that *PIK3R1* is differentially expressed in OA affected cartilage, these results identify *PIK3R1* as the most likely causal gene. Another possibility is that not *PIK3R1* but rather a long-non-coding RNA (lncRNA), lnc-PIK3R1-4:1, is causal, since a variant in LD with the lead SNP is located in the predicted transcription start site of this lncRNA potentially affecting its expression. Although conserved in mice and zebrafish, thus far no function has been ascribed to this lncRNA [34].

We confirmed a locus previously associated with cartilage thickness, the *DOT1L* locus. Our identified SNP, *rs11880992* is in high LD with the previously reported SNP *rs129827744*, and both are associated with cartilage thickness and hip OA [1]. Despite the previously presented suggestive evidence for involvement of DOT1L in chondrogenic differentiation, *DOT1L* did not receive a high score in our systematic prioritization study; the gene *GADD45B*, located in the region 500Kb downstream of the lead SNP, received a similar score. GADD45B is a transcriptional co-factor for C/EBP-β, a master regulator of chondrocyte differentiation [35]. Thus, it remains unclear which gene or genes in this locus contribute to the cartilage phenotype. Further research is needed to determine whether *DOT1L* is the true causal gene in this locus.

Our analyses suggest that the majority of prioritized genes in hip OA associated loci are involved in cartilage and bone developmental pathways; including *TGFA*, *RUNX2*, *FGFR3*, *PTHLH*, *COL12A1* and others that seem to affect bone and/or cartilage development such as *PIK3R1* and *KMT2A* We hypothesize that the mJSW and OA associated variants influence gene expression regulation. The dysregulation of these genes and mechanisms during development may, later in life, result in an increased risk for OA.

The identified mJSW SNPs are associated with hip OA, but not with knee OA. We have analysed the identified SNPs also for association with knee OA in the TREAT-OA meta-analysis dataset [36], but found no association. This observation fits in the overall finding that many of the identified genetic loci for OA seem to be site-specific [37], and support the hypothesis that the aetiology of OA is different in each joint. Nevertheless, this observation can still be a result of low power in the GWAS studies that have been done for OA till now [38], and final conclusions on this aspect cannot be drawn at this point.

This is the first report linking TGFA to human OA most likely by affecting mJSW. It may serve as a new target for future therapies. We have identified multiple mJSW associated loci which have previously been associated with other bone and cartilage related phenotypes such as bone mineral density and height, displaying a possible pleiotropic effect for the analysed traits. It will be important to understand how mJSW and OA associated variants can affect the developmental processes that regulate morphometry of the hip joint, including the formation of articular cartilage. Therefore further expression and functional studies are warranted of genes identified to be associated with hip OA phenotypes.

## Materials and Methods

### Ethics statement

The participating studies were approved by the medical ethics committees of all participating centres, and all participants gave their written informed consent before entering the study

### Discovery GWAS, replication and meta-analysis

We conducted genome-wide association studies of mJSW for each cohort of the discovery stage: Rotterdam Study I (RS-I), Rotterdam Study II (RS-II), TwinsUK, SOF and MrOS using standardized age-, gender and population stratification (four principal components) adjusted residuals from linear regression. Cohort description and details of the single GWAS studies are given in S1 Text and S1 Table. The 6 cohorts used in the discovery stage were combined in a joined meta-analysis using inverse variance weighting with METAL [39]. Genomic control correction was applied to the standard errors and P-values before meta-analysis. SNPs with a P value < = 5×10−^6^ were selected for replication. The top SNPs for each independent locus were taken for replication in seven studies: the Genetics of Osteoarthritis and Lifestyle (GOAL) study, the Chingford study, CHECK (Cohort Hip & Cohort Knee), Genetics osteoARthritis and progression (GARP) study, the Genetics of Generalized Osteoarthritis (GOGO), the Johnston County Osteoarthritis Project (JoCo) and additionally the Nottingham OA case-control study for association with Hip OA (see Supplemental material for detailed information of the cohorts). Association of the SNPs with mJSW was additionally adjusted for height to test its independence. Secondary analyses included: association of the top SNPs with hip OA using logistic regression analysis (age and gender adjusted and by study centres an/or relatedness when it was pertinent). We used conditional analyses to investigate whether there are any independent signals in the identified associated loci, which were implemented using GCTA-COJO analysis [40].

### Phenotype description of minimal Joint Space width (mJSW)

The mJSW was assessed at pelvic radiographs in anteriorposterior position. The mJSW was measured in mm, along a radius from the center of the femoral head, and defined as the shortest distance found from the femoral head to the acetabulum. Within the Rotterdam Study, we used a 0.5 mm graduated magnifying glass laid directly over the radiograph to measure the minimal joint space width of the hip joints [41]. Within SOF and MrOS, a handheld caliper and reticule was used to measured mJSW to the nearest 0.1mm between the acetabular rim and proximal head of the femur [42]. For CHECK, mJSW was measured semi-automatic with the Software tool HOLY [43].

### Phenotype description of radiographic hip OA

Radiographic hip OA was defined in the RS-I, RS-II, RSIII, Twins-UK, Chingford, and JoCo studies using Kellgren and Lawrence (K/L) scores. Hip OA cases were defined as a K/L score ≥ 2 on either side of the hip or THR due to OA. Hip OA controls were defined as no THR for OA and K/L score ≤ 1 and JSN ≤ 1. In MrOS and SOF cohorts, radiographic hip OA case-control was defined by a modified Croft grade, as previously described [44], where cases were defined as a Croft score ≥ 2 on either side of the hip or THR due to OA and controls were defined as a Croft score ≤ 1 on both sides of the hip and no THR. Hip OA cases in the GOAL and Nottingham OA studies were defined by having THR, and controls were radiographically free of hip OA, as previously described [45]. In GARP, hip osteoarthritis was defined as pain or stiffness in the groin and hip region on most days of the preceding month in addition to femoral or acetabular osteophytes or axial joint space narrowing on radiography or prosthesis due to osteoarthritis. In GOGO, hip OA was defined as KL grade > = 2, or minimal joint space width < = 2.5 mm, or the combination of joint space narrowing grade > = 2 and any osteophyte of grade > = 1, or history of joint replacement for OA. In JoCo, hip OA cases were defined as KL grade > = 2 or THR in at least one hip. Hip OA controls were defined as KL grade < = 1 in both hips.

### Gene prioritization analysis

We have used several available tools and publicly available databases to prioritize genes in known and newly discovered osteoarthritis associated regions. Locus gene sets were constructed by taking a region of 500 Kb upstream and 500Kb downstream of the lead SNP of that locus. We analysed 152 genes in 13 independent loci associated with minimal joint space width in the hip joint (mJSW) for 7 loci, hip OA for 4 loci, total joint replacement (TJR) for 1 locus and total hip replacement (THR) for 1 locus [2]. We analysed the following biological evidence for each gene at all loci; Nearest located genes: Taken from the UCSC genome browser, GRCh37/hg19 [46]. DEPICT gene prioritization: Data-driven Expression-Prioritized Integration for Complex Traits, a novel tool designed to identify the most likely causal gene in a given locus and to gene sets that are enriched in the genetic associations [21]. DEPICT was used to prioritize genes in a 1 MB region around the found SNPs that were significant associated with the osteoarthritis phenotype, taking a region of 500 Kb upstream and 500Kb downstream of the lead SNP of that locus. Gene prioritization analysis was performed to directly investigate functional similarities among genes from different associated regions, significance was defined by false discovery rate (FDR ≤ 5%). GRAIL gene prioritization: Gene Relationships Across Implicated Loci (GRAIL), was used to determine connectively between genes across OA implicated loci based on literature associations [22]. A GRAIL analysis was performed on 10 independent OA associated loci, based on existing literature in PubMed till August 2014. Mouse gene expression and phenotype: For each investigated gene, expression in mouse bone and/or cartilage tissue during several developmental stages as well as for adult tissue was determined using data from the Jackson lab database (http://www.informatics.jax.org/). In addition mouse phenotype data was also obtained for each gene. OMIM phenotype: Using the Online Mendelian Inheritance in Man (OMIM) database we examined which genes were involved in abnormal skeletal growth syndromes when mutated (http://omom.org). Expression quantitative trait loci: eQTL information was taken from the Blood eQTL browser (http://genenetwork.nl/bloodeqtlbrowser/) and the eQTL browser (http://www.ncbi.nlm.nih.gov/projects/gap/eqtl/index.cgi) using the lead SNP in each locus [20]. Non-synonymous variants: Last we determined if there were any nonsynonymous variants in LD (r^2^>0.06) with the lead SNP of a locus, using HaploReg V2 and the SNP Annotation and Proxy Search (SNAP) tools [47,48]. For each gene we assigned a score based on equally weighted lines of evidence.

### Human cartilage gene expression

We have used cartilage samples from the RAAK study to study gene expression in preserved and affected cartilage from individuals undergoing joint replacement [19]. The ongoing Research Arthritis and Articular Cartilage (RAAK) study is aimed at the biobanking of blood, joint materials (cartilage, bone and where available ligaments of knees and hips) and bone marrow stem cells (hip joints only) of patients and controls in the Leiden University Medical Center and collaborating outpatient clinics in the Leiden area. At the moment of collection (within 2 hours following surgery) tissue was washed extensively with phosphate buffered saline (PBS) to decrease the risk of contamination by blood, and cartilage was collected of the weight-bearing area of the joint. Cartilage was classified macroscopically and collected separately for macroscopically OA affected and preserved regions. Classification was done according to predefined features for OA related damage based on color/whiteness of the cartilage, based on surface integrity as determined by visible fibrillation/crack formation, and based on depth and hardness of the cartilage upon sampling with a scalpel. During collection with a scalpel, care was taken to avoid contamination with bone or synovium. Collected cartilage was snap frozen in liquid nitrogen and stored at -80°C prior to RNA extraction. Tissues have been stored tailored to apply staining and immunohistochemistry (IHC). Furthermore, DNA and RNA have been isolated from the preserved and affected areas of the respective tissues in order to apply genetic, transcriptomic and epigenomic profiling with respect to the OA pathophysiological process.

After *in vitro* transcription, amplification, and labeling with biotin-labeled nucleotides (Illumina TotalPrep RNA Amplification Kit) Illumina HumanHT-12 v3 microarrays were hybridized. Sample pairs were randomly dispersed over the microarrays, however each pair was measured on a single chip. Microarrays were read using an Illumina Beadarray 500GX scanner and after basic quality checks using Beadstudio software data were analyzed in R statistical programming language. Intensity values were normalized using the “rsn” option in the Lumi-package and absence of large scale between-chip effects was confirmed using the Globaltest-package in which the individual chip numbers were tested for association to the raw data. After removal of probes that were not optimally measured (detection *P* >0.05 in more than 50% of the samples) a paired t-test was performed on all sample pairs while adjusting for chip (to adjust for possible batch effects) and using multiple testing correction as implemented in the “BH” (Benjamini and Hochberg) option in the Limma-package. Analyses for differential expression between OA and healthy and between preserved and healthy cartilage was performed likewise, adjusting in addition for sex and for age.

### Exome sequencing

Exome sequencing was performed in 2628 individuals from the Rotterdam Study the average mean coverage was 55x, corresponding to approximately 80% of the targeted regions covered by at least 20 reads. The exome sequencing was performed in house (HuGe-F, www.glimDNA.org). Details of the technical procedure and variant calling are given in S2 Text. We tested the exome variants for association with mJSW and/or hip OA in two ways. Each individual variant was tested for association with mJSW using the single variant option within RV-test, while adjusting for age and sex. In addition, we did a burden test for each of the selected genes by using SNP-set kernel association test (SKAT-O). SKAT aggregates individual score test statistics of SNPs in a SNP set and computes SNP-set level p-values for a gene [23].

### Visualization of the regulatory landscape of mJSW associated loci

For each of the top mJSW GWAS associated SNPs the LD region was determined using the 1000G Phase 1 population using the Haploreg tool [47]. The LD threshold was set at r2≥0.8. For each of these SNPs it was determined if the variant was located in a potential enhancer region using the Roadmap consortium reference epigenomes data set [27]. Heatmaps were constructed by calculating the percentage of variants in LD with the top mJSW GWAS found SNP located in enhancer regions as defined by the Roadmap epigenome chromatin states. The reference epigenomes were downloaded from the official data portal accompanying [27]. Reference epigenome data was used from mesenchymal stem cell derived chondrocyte cultured cells, Osteoblast, Bone marrow derived cultured mesenchymal stem cells, K562, HUVEC, HeLA and NHEK cells. Reference epigenomes were chromatin state models based on ChIPseq data of 5 core histone marks (H3K4me3, H3K4me1, H3K36me3, H3K27me3, H3K9me3) and an additional H3K27ac histone mark, the Roadmap expanded 18-state model.

ChIPseq data of mesenchymal stem cell derived chondrocyte cultured cells, and bone marrow derived cultured mesenchymal stem cells were generated by the NHI roadmap epigenomics project [28]. ChIPseq data of, Osteoblast, K562, HUVEC, HeLA and NHEK cells were generated by the ENCODE consortium [26]. All data and annotation tracks were downloaded through the UCSC genome browser table tool. Visualization of all ChIPseq annotation and roadmap full epigenomes tracks was done through the UCSC genome browser on GRCh37/hg19. Heatmaps were plotted in R using the CRAN software packages gplots and RcolorBrewer. Enrichment was calculated according to methods described in Trynka et al [25].

## Supporting Information

S1 Fig Interquantile-quantile plot for the analysis of mJSW in the discovery cohorts. (TIF)

S1 Table Baseline characteristics of the studies included in the analyses of minimal Joint Space Width. (XLSX)

S2 Table Quality control and inclusion criteria. (XLSX)

S3 Table Analysed SNPs in discovery and replication cohorts. (XLSX)

S4 Table Results from the GCTA analysis on chromosome 6. (XLSX)

S5 Table Results from the GRAIL analysis. (XLSX)

S6 Table Results from the DEPICT analysis. (XLSX)

S7 Table Human and Mouse Phenotypes, gene expression in healthy tissue (human, mouse). (XLSX)

S8 Table eQTL analysis of hipOA related SNPs. (XLSX)

S9 Table Non-synonymous variants linked to the GWAS SNP. (XLSX)

S10 Table Gene expression in human cartilage tissue (differential expression) (XLSX)

S11 Table Association results of all Exonic variants (identified with Exome-seq) and mJSW. (XLSX)

S12 Table Results of multivariate analyses testing the joint effect of GWAS hits and exome variants on mJSW. (XLSX)

S13 Table Variants in linkage disequilibrium with rs11880992 which are located in regulatory regions. (XLSX)

S14 Table Variants in linkage disequilibrium with rs496547 which are located in regulatory regions. (XLSX)

S15 Table Variants in linkage disequilibrium with rs104948155 which are located in regulatory regions. (XLSX)

S16 Table Variants in linkage disequilibrium with rs2862851 which are located in regulatory regions. (XLSX)

S17 Table Variants in linkage disequilibrium with rs2206662 which are located in regulatory regions. (XLSX)

S18 Table Variants in linkage disequilibrium with rs10471753 which are located in regulatory regions. (XLSX)

S1 Text Description of cohorts. (DOCX)

S2 Text Description of Exome sequencing and variant calling. (DOCX)
