# Supplementary figures and images for: Novel Genetic Variants for Cartilage Thickness and Hip Osteoarthritis
Source: PLoS Genet. 2016 Oct 4;12(10):e1006260. doi: 10.1371/journal.pgen.1006260 (PMC5049763; doi:10.1371/journal.pgen.1006260)

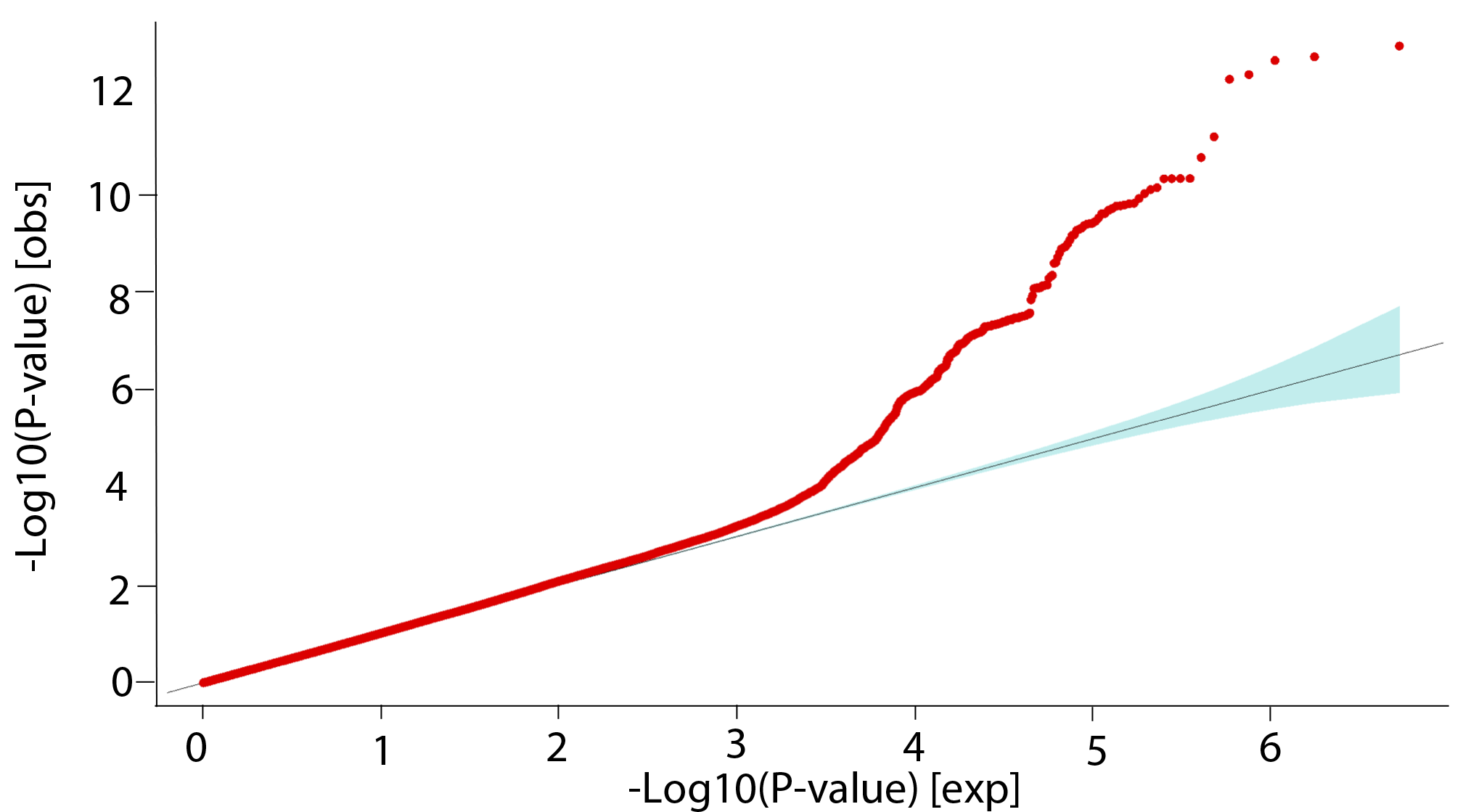

Supplement: S1 Fig — (TIF) [file pgen.1006260.s001.tif]
