# Supplementary material for: Novel Genetic Variants for Cartilage Thickness and Hip Osteoarthritis
Source: PLoS Genet. 2016 Oct 4;12(10):e1006260. doi: 10.1371/journal.pgen.1006260 (PMC5049763; doi:10.1371/journal.pgen.1006260)
Supplement: S1 Text — (DOCX) [file pgen.1006260.s020.docx]

**S1 text: Description of cohorts**

All participating cohorts are described in detail below. The participating studies were approved by the medical ethics committees of all participating centres, and all participants gave their written informed consent before entering the study.

**Discovery Cohorts Description, Genotyping & QC**

**Rotterdam Study.** The Rotterdam study is a population-based prospective cohort study on-going since 1990 to study determinants of chronic disabling disease ([1](#_ENREF_1)). The Rotterdam Study I (RS-I) is the first cohort of 7,983 persons living in the Ommoord district of Rotterdam in the Netherlands. All subjects were aged 55 years and older and recruitment started in 1990. The Rotterdam Study II (RS-II) started in 1999 when 3,011 participants moved into the study since they became 55 years of age or moved into the study district. Genotyping of the samples in the discovery cohorts (RS-I and RS-II) was carried out with the Illumina HumanHap 550v3 Genotyping BeadChip. The Beadstudio GenCall algorithm was used for genotype calling and quality control procedures, as described previously ([2](#_ENREF_2)). The following quality control inclusion filters were applied: call rate ≥97.5%, MAF ≥1%, *P* for Hardy-Weinberg equilibrium <1 × 10^−6^ **(Table S1** for details on quality control and exclusion criteria**)**. The total number of genotyped SNPs that passed these filters was 512,349 for RS-I and 466,389 for RS-II. Imputation was done with reference to HapMap release 22 CEU using the maximum likelihood method implemented in MACH (<http://www.sph.umich.edu/csg/abecasis/MACH/index.html>). Analysis of imputed genotype data accounted for uncertainty in each genotype prediction by using the dosage information from MACH. For this analysis, MACH2QTL, was used via GRIMP ([3](#_ENREF_3)), which uses genotype dosage value (0–2, continuous) as a predictor in a linear regression framework. We included only imputed SNPs that had a good imputation quality leaving a total of 2,451,799. The summary statistics of RS-I and RS-II were meta-analysed using METAL applying inverse-variance methodology assuming fixed effects with Cochran's Q and I2 metrics used to quantify between-study heterogeneity (www.sph.umich.edu/csg/abecasis/metal).

The medical ethics committee of Erasmus University Medical School approved the study and written informed consent was obtained from each participant.

**TwinsUK.** The TwinsUK study participants were white monozygotic and dizygotic twin pairs from the TwinsUK adult twin registry, a group used to study the heritability and genetics of age-related diseases (4). These unselected twins were recruited from the general population through national media campaigns in the United Kingdom. All samples from the TwinsUK cohort for this study were genotyped with the HumanHap610Q (Illumina). The following quality control filters were applied: call rate ≥98%, MAF ≥ 1%, *P* for Hardy-Weinberg equilibrium ≥1 × 10^−6^ (Table S1). The total number of genotyped SNPs that passed these filters was 598,207 SNPs. Imputation was done with reference to HapMap release 22 CEU using the IMPUTE software package (v2) ([5](#_ENREF_4)). The St. Thomas' Research Ethics Committee approved the study (EC96/439 TwinsUK), and all participants provided informed written consent.

**SOF.** The Study of Osteoporotic Fractures (SOF) is a prospective multicenter study of risk factors for vertebral and non-vertebral fractures (6). The cohort at the baseline visit is comprised of 9,704 community dwelling women 65 years old or older recruited from populations-based listings in four U.S. areas: Baltimore, Maryland; Minneapolis, Minnesota; Portland, Oregon; and the Monongahela Valley, Pennsylvania. For further details on inclusion criteria, please see. Institutional Review Boards at each site approved the study. Written informed consent was present for all participants. Among the 9704 SOF participants enrolled at the baseline visit, 6795 participants provided blood samples and consented to genetic testing, and DNA samples from 3924 of these participants had sufficient quantity as assayed by Picogreen and underwent whole genome genotyping at the Broad Institute.

The Illumina HumanOmni1 Quad genotyping array, containing 1,016,423 probes, was used for whole-genome genotyping in MrOS and SOF samples. Quality control procedures for SNPs and samples are described in Table S1. In addition to the SNP filters described in Table S1, SNPs with GenTrain scores <0.6 or cluster separation scores <0.4 were excluded. Additional samples were excluded based on: (1) genotypic sex mismatch using X and Y chromosome probe intensities, (2) relatedness among genotyped samples using the kinship coefficient that estimates probability that alleles are identical-by-descent, and (3) gross chromosomal abnormalities detected using the LogR Ratio and B allele frequency. Among the 3924 SOF samples that underwent whole-genome genotyping, 3682 samples had acceptable call rates. Among the 3924 SOF samples that underwent whole-genome genotyping, 3682 samples had acceptable call rates, and of these, 4 were removed due to relatedness and 53 were removed due to gross chromosomal abnormalities, leaving 3625 SOF samples with whole genome genotyping data that passed QC. Subjects with the following criteria were then excluded from the present analysis: Paget’s Disease, rheumatoid arthritis, non-European ancestry, prevalent hip fracture, total hip replacement (THR) for fracture or other non-OA causes.

**MrOS.** The Osteoporotic Fractures in Men Study (MrOS) is a multi-center prospective, longitudinal, observational study of risk factors for vertebral and all non-vertebral fractures in older men, and of the sequelae of fractures in men (7). The MrOS study population at the baseline visit consists of 5,994 community dwelling, ambulatory men aged 65 years or older from six communities in the United States. Approval of the conduct of the MrOS study was obtained from the institutional review boards of the participating clinics and written informed consent was obtained from all study participants.

Among the 5994 MrOS participants enrolled at the baseline visit, 5527 participants provided blood samples, had DNA extracted, and consented to genetic testing. Among the 5527 MrOS DNA samples, 5485 had sufficient DNA quantity and DNA solution volume and underwent whole-genome genotyping. Among the 5485 MrOS samples that underwent whole-genome genotyping, 5168 samples had acceptable call rates. Among these 5168 MrOS samples, 1 was removed due to relatedness and 37 were removed due to gross chromosomal abnormalities, leaving 5130 MrOS samples with whole genome genotyping data that passed QC. Subjects with the following criteria were then excluded from the present analysis: Paget’s Disease, rheumatoid arthritis, non-European ancestry, prevalent hip fracture, total hip replacement (THR) for fracture or other non-OA causes. In SOF and MrOS imputation was done with reference to HapMap release 22 CEU using the MACH/minimac software.

**Replication Cohorts: description, Genotyping & QC**

**GOAL**. The Genetics of Osteoarthritis and Lifestyle (GOAL) study and the Nottingham OA case-control study have been previously described (8). Hip OA cases were recruited from hospital orthopaedic surgery lists in Nottingham. Cases had been referred to the hospital with symptomatic, clinically severe hip or knee OA and the majority had undergone unilateral or bilateral THR or TKR within the previous 5 years. Pre-operative knee or pelvis radiographs were examined to confirm the diagnosis. Subjects were excluded if they had another major arthropathy, Paget’s disease, overt child hip disease, THR due to trauma or terminal illness. Approval for recruitment of knee and hip OA cases was obtained from the research ethics committees of Nottingham City Hospital and North Nottinghamshire. Controls were age-matched individuals from the same catchment area free from radiographic OA and over the age of 55. Genomic DNA was extracted from peripheral blood leukocytes of affected individuals and controls using standard protocols. Genotyping was carried out by Kbioscience Ltd. SNPs were genotyped using the KASPar chemistry, which is a competitive allele-specific polymerase chain reaction (PCR) SNP genotyping system using fluorescence resonance energy transfer (FRET) quencher cassette oligos.

**Chingford.** The Chingford study is a prospective population-based longitudinal cohort, which includes women derived from the age/sex register of a large general practice in North London (9). Genomic DNA was extracted from peripheral blood leukocytes of affected individuals and controls using standard protocols. Genotyping was done on xx individuals using…..

For those SNPs that were not genotyped with the array, proxy SNPs were analysed. Information about the proxy SNPs is given in the table beneath.

| SNPID | PROXY | Proxy | Distance | R_sq | Dprime |
| --- | --- | --- | --- | --- | --- |
| rs10948155 | rs10948155 | NA | NA | NA | NA |
| rs11045356 | rs11045356 | NA | NA | NA | NA |
| rs11880992 | rs12974139 | Y | 7602 | 0,873 | 1 |
| rs2862851 | rs1807968 | Y | 4541 | 1 | 1 |
| rs10495106 | rs2133045 | Y | 54786 | 1 | 1 |
| rs2236995 | rs2236995 | NA | NA | NA | NA |
| rs2703529 | rs2703529 | NA | NA | NA | NA |
| rs4837613 | rs4836732 | Y | 42830 | 0,807 | 0,929 |
| rs6429001 | rs6429001 | NA | NA | NA | NA |
| rs6437120 | rs6737672 | Y | 1719 | 0,935 | 1 |
| rs13148031 | rs720485 | Y | 2250 | 1 | 1 |
| rs717433 | rs7269743 | Y | 1421 | 1 | 1 |
| rs10471753 | rs7726943 | Y | 371 | 1 | 1 |
| rs2061109 | rs814487 | Y | 29880 | 0,84 | 1 |
| rs7739938 | rs9389685 | Y | 13429 | 1 | 1 |
| rs12206662 | Rs12210292 | Y | 450928 | 021 | 1NA |
| rs6592847 | Rs7358419 | Y | 4831 | 0,57 | 1 |
| rs496547 | Rs487728 | Y | 45032 | 0.69 | 0.84 |

**GARP.** Genetics ARthrosis and Progression study (GARP). The GARP study (10) is aimed at identifying determinants of osteoarthritis susceptibility and progression. The study is based on sibships of white Dutch ancestry with predominantly symptomatic osteoarthritis at multiple sites. Patients (probands) aged between 40 to 70 years with symptomatic osteoarthritis in the hands, knees, or hips—diagnosed by rheumatologists, orthopaedic surgeons, and general practitioners in Leiden, The Hague, Delft, Haarlem, and Amsterdam—were informed of the ongoing study by mail. Interested probands were subsequently sent a mailed questionnaire about demographic data, medical history, symptoms and signs of osteoarthritis, and family history of osteoarthritis. Subsequently probands with osteoarthritis at multiple sites and with a positive family history were requested to introduce a sibling “with joint complaints,” who was also sent a questionnaire. After obtaining informed consent, all sibships underwent a physical examination and were assessed by a single medical doctor (NR) at the outpatient clinic. Patients with secondary osteoarthritis and familial syndromes with a Mendelian inheritance pattern were excluded. Osteoarthritis developing under the following conditions was considered secondary: major congenital or developmental diseases and bone dysplasias; major local factors such as severe scoliosis and hypermobility; certain metabolic diseases associated with joint disease such as haemochromatosis and Wilson’s disease; inflammatory joint diseases such as rheumatoid arthritis; other bone diseases such as Paget’s disease and osteochondritis; intra-articular fracture. The GARP study was genotyped using Illuminia Infinium HD Human660W-Quad Beadchips (Illumin, San Diego, CA, USA) (ref). Subsequently, imputation was performed with Hapmap2 using IMPUTE.

**JoCo.** The Johnston County Osteoarthritis Project (JoCo) is an ongoing population-based prospective cohort study of the occurrence of knee and hip OA in African Americans and Caucasians in Johnston County, North Carolina (11). This project was designed as a long-term study of ethnic differences in OA occurrence and progression. The samples were collected from six townships among the 17 townships in Johnston County because they contained the largest proportion of African American residents. The study was approved by the Institutional Review Boards of the University of North Carolina Schools of Medicine and Public Health and the Centers for Disease Control and Prevention. All partic- ipants gave written informed consent at the time of recruitment.

The participants were initially recruited at the baseline between 1990 and 1997 and were followed up between 1999 and 2004. Additional new individuals were enrolled in 2003–2004 to enrich the sample for African Americans and younger individuals who were deliberately targeted for inclusion. A total of 2583 participants from the Johnston County Cohort were selected from the total study population for genotyping. Participants selected for inclusion in the present association study were Caucasians of European ancestry (68% of genotyped cohort) of both sexes (35% men).

**GOGO**. The Genetics of Generalized Osteoarthritis (GOGO) Study is a collaborative study involving seven academic sites, five in the United States (US) and two in the UK. Recruitment began in all sites in 2000 and was completed in 2002. Participants were recruited inform Rheumatology clinics, hospital databases of OA patients, pre-existing OA cohort, ad from community, by advertisements. A qualifying family consisted of a least two siblings with self-reported Caucasian ethnicity who fulfilled clinical GOGO and OA criteria (bony enlargements of >=3 joints distributed bilaterally, including bone enlargement of at least one DIP joint, and no more than three swollen metacarpophalangeal joints). In a family the first individual that met clinical GOGO and OA criteria was designated the proband. A total of 1145 families were recruited (12). From the participants, whole blood 5 ml for sera and 23 ml for plasma and DNA extraction was collected. For those SNPs that were not genotyped with the array, proxy SNPs were analysed. Information about the proxy SNPs is given in the table beneath.

| SNPID | PROXY | Proxy | Distance | R_sq | Dprime |
| --- | --- | --- | --- | --- | --- |
| rs10948155 | rs10948155 | NA | NA | NA | NA |
| rs11045356 | rs11045356 | NA | NA | NA | NA |
| rs11880992 | rs12974139 | Y | 7602 | 0,873 | 1 |
| rs2862851 | rs1807968 | Y | 4541 | 1 | 1 |
| rs10495106 | rs2133045 | Y | 54786 | 1 | 1 |
| rs2236995 | rs2236995 | NA | NA | NA | NA |
| rs2703529 | rs2703529 | NA | NA | NA | NA |
| rs4837613 | rs4836732 | Y | 42830 | 0,807 | 0,929 |
| rs6429001 | rs6429001 | NA | NA | NA | NA |
| rs6437120 | rs6737672 | Y | 1719 | 0,935 | 1 |
| rs13148031 | rs720485 | Y | 2250 | 1 | 1 |
| rs717433 | rs7269743 | Y | 1421 | 1 | 1 |
| rs10471753 | rs7726943 | Y | 371 | 1 | 1 |
| rs2061109 | rs814487 | Y | 29880 | 0,84 | 1 |
| rs7739938 | rs9389685 | Y | 13429 | 1 | 1 |
| rs12206662 | None | NA | NA | NA | NA |
| rs6592847 | None | NA | NA | NA | NA |
| rs496547 | None | NA | NA | NA | NA |

**CHECK**. It is a multi-centre cohort formed by the Dutch Arthritis Association (DAA) with over 1000 participants with pain of hip and/or knee expected to develop knee and or hip OA: CHECK (Cohort Hip & Cohort Knee) (13). The objective of CHECK is to study the course of complaints, the mechanisms that cause joint damage, and to identify markers for diagnosis and prognosis, as well as to identify prognostic factors that predict and explain the course of OA. On entry, all participants had pain of knee or hip, were aged 45-65 years. They had not yet consulted their physician for these symptoms, or the first consultation was within 6 months before entry. Any other pathological condition that could explain the existing complaints was excluded (e.g. other rheumatic disease, previous hip or knee joint replacement, congenital dysplasia, osteochondritis dissecans, intra-articular fractures, septic arthritis, Perthes' Disease, ligament or meniscus damage, plica syndrome, Bakers cyste). Participants visit the hospital every year and the following data and materials are gathered. The study was approved by the medical ethics committees of all participating centres, and all participants gave their written informed consent before entering the study. mJSW was detemined on X-rays using a previously published software tool([14](#_ENREF_5)). Blood samples were collected at baseline and de-novo genotyping of the selected SNPs for replication was carried out with

Sequenom iPLEX and Taqman Allelic Discrimination. Genomic DNA was extracted from samples of peripheral venous blood according to standard procedures. 4 ng genomic DNA was dispensed into 384-wells plates using a Caliper Sciclone ALH3000 pipetting robot (Caliper LS, Mountain View, CA, USA). For this, sequences containing the SNP site and at least 100 bp of flanking sequence on either side of the SNP were used. Briefly, 2 ng genomic DNA was amplified in a 5 ul reaction containing 1 × Taq PCR buffer (Sequenom), 2 mM MgCl2, 500 uM each dNTP, 100 nM each PCR primer, 0.5 U Taq (Sequenom).

In silico replication was performed in GOGO, Chingford, GARP, and JoCo studies (Table S2). De novo SNP genotyping was performed in the GOAL, CHECK and Nottingham OA case-control studies (Table S2).

1. Hofman A, van Duijn CM, Franco OH, Ikram MA, Janssen HL, Klaver CC, et al. The Rotterdam Study: 2012 objectives and design update. Eur J Epidemiol. 2011 Aug;26(8):657-86.

2. Richards JB, Rivadeneira F, Inouye M, Pastinen TM, Soranzo N, Wilson SG, et al. Bone mineral density, osteoporosis, and osteoporotic fractures: a genome-wide association study. Lancet. 2008 May 3;371(9623):1505-12.

3. Estrada K, Abuseiris A, Grosveld FG, Uitterlinden AG, Knoch TA, Rivadeneira F. GRIMP: a web- and grid-based tool for high-speed analysis of large-scale genome-wide association using imputed data. Bioinformatics. 2009 Oct 15;25(20):2750-2.

4. Spector RD, Williams FM. The UK Adult Twin Registry (TwinsUK). Twin Res.Hum. Genet. 2006 Dec;9(6): 899-906.

5. Howie BN, Donnelly P, Marchini J. A flexible and accurate genotype imputation method for the next generation of genome-wide association studies. PLoS genetics. 2009 Jun;5(6):e1000529.

6. Black DM, PAlermo L, Nevitt MC, Genant HK, Epstein R, San Valentin R, Cummings SR Comparison of methods for defining prevalent vertebral deformities: the Study of Osteoporotic Fracture**s.** J Bone Miner Res. 1995 Jun;10(6):890-902.

7. Orwoll E, Blank JB, Barrett-Connor E, Cauley J, Cummings S, Ensrud K, Lewis C, Cawthon PM, Marcus R, Marshall LM, McGowan J, Phipps K, Sherman S, Stefanick ML, Stone K. Design and baseline characteristics of the osteoporotic fractures in men (MrOS) study--a large observational study of the determinants of fracture in older men. Contemp Clin Trials. 2005 Oct;26(5):569-85.

8. Valdes AM, McWilliams D, Arden NK, Doherty SA, Wheeler M, Muir KR, Zhang W, Cooper C, Maciewicz RA, Doherty M. Involvement of different risk factors in clinically severe large joint osteoarthritis according to the presence of hand interphalangeal nodes Arthritis Rheum. 2010 Sep;62(9):2688-95.

9. Hart DJ, Doyle DV, Spector TD. Association between metabolic factors and knee osteoarthritis in women: the Chingford Study. J Rheumatol. 1995 Jun;22(6):1118-23

10 Riyazi N1, Meulenbelt I, Kroon HM, Ronday KH, Hellio le Graverand MP, Rosendaal FR, Breedveld FC, Slagboom PE, Kloppenburg M. Evidence for familial aggregation of hand, hip, and spine but not knee osteoarthritis in siblings with multiple joint involvement: the GARP study. Ann Rheum Dis. 2005 Mar;64(3):438-43.

11. Elliott AL, Kraus VB, Fang F, Renner JB, Schwartz TA, Salazar A, Huguenin T, Hochberg MC, Helmick CG, Jordan JM. Joint-specific hand symptoms and self-reported and performance-based functional status in African Americans and Caucasians: The Johnston County Osteoarthritis Project. Ann Rheum Dis. 2007 Dec;66(12):1622-6

12. Jordan JM, Doherty M, Wilson AG, Moskowitz R, Hochberg M, Loeser R, Hooper M, Renner JB, Crane MM, Hastie P, Sundseth S, Atif U. The Genetics of Generalized Osteoarthritis (GOGO) study: study design and evaluation of osteoarthritis phenotypes. Osteoarthritis Cartilage. 2007 Feb;15(2):120-7.

13. Wesseling J, Dekker J, van den Berg WB, Bierma-Zeinstra SM, Boers M, Cats HA, Deckers P, Gorter KJ, Heuts PH, Hilberdink WK, Kloppenburg M, Nelissen RG, Oosterveld FG, Oostveen JC, Roorda LD, Viergever MA, ten Wolde S, Lafeber FP, Bijlsma JW. CHECK (Cohort Hip and Cohort Knee): similarities and differences with the Osteoarthritis Initiative. Ann Rheum Dis. 2009 Sep;68(9):1413-9.

14. Kinds MB, Vincken KL, Vignon EP, ten Wolde S, Bijlsma JW, Welsing PM, et al. Radiographic features of knee and hip osteoarthritis represent characteristics of an individual, in addition to severity of osteoarthritis. Scand J Rheumatol. 2012 Mar;41(2):141-9.
