# Supplementary material for: Novel Genetic Variants for Cartilage Thickness and Hip Osteoarthritis
Source: PLoS Genet. 2016 Oct 4;12(10):e1006260. doi: 10.1371/journal.pgen.1006260 (PMC5049763; doi:10.1371/journal.pgen.1006260)
Supplement: S2 Text — (DOCX) [file pgen.1006260.s021.docx]

**S2 text: Description of Exome sequencing and variant calling**

**Exome sequencing**

Genomic DNA of participants were prepared from blood and fragmented into 200-400 bp fragments using Covaris Adaptive Focused Acoustics (AFA) shearing according to the manufacturer’s instructions (Covaris, Inc., Woburn, MA). Illumina TruSeq DNA Library preparation (Illumina, Inc., San Diego, CA) was performed on a Caliper Sciclone NGS workstation (Caliper Life Sciences, Hopkinton, MA), followed by exome capture using the Nimblegen SeqCap EZ V2 kit (Roche Nimblegen, Inc., Madison, WI). This capture targets 44Mb of exonic regions covering 30,246 coding genes, 329,028 exons and 710 miRNAs. Paired-end 2 x 100 sequencing was performed at 6 samples per lane on Illumina HiSeq2000 sequencer using Illumina TruSeq V3 chemistry.

***Data processing***

High quality reads were demultiplexed and aligned to the human reference genome hg19 (UCSC, Genome Reference Consortium GRCh37) using the Burrows-Wheeler alignment tool (BWA version 0.7.3a (1). After indel realignment and base quality score recalibration using the Genome Analysis ToolKit (GATK version 2.7.4 (2) and masking of duplicates (Picard Tools version 1.90 (3), per sample SNV and Indel calling was performed using HaplotypeCaller v3.1.1 (GATK).

***Gathering Sample QC Metrics***

Sample QC metrics were obtained using GATKs DepthofCoverage and VariantEvaluation modules. Background noise levels were estimated and corrected for using the verifyBAMid tool and the “contamination fraction” option in GATKs HaplotypeCaller. Concordance with SNP array data (HumanHap550-Quad+ BeadChip, Illumina) was determined by comparing heterozygous calls on the array with the exome sequencing data per sample.

***Technical and biological sample QC***

We excluded all samples from subsequent analysis when the mean depth of coverage was less than 20x, the freemix estimate was higher than 13% or samples with a genotype concordance below 98% (n=333). The gVCF files for the remaining samples were combined per 300 samples using GATKs combineGVCFs and genotyped using GATKs genotypGVCF module. SNVs and Indels were separated and subjected to GATKs Variant-Quality Score Recalibration. Finally, VariantEvaluation was performed on each sample to identify samples with poor quality call sets (ie: many false positives).

During biological QC we excluded all samples where the observed number of SNVs, het/hom ratio or Ti/Tv ratio were 4 standard deviations or more from the mean (n=34). Additionally, samples with any of these values between 2 and 4 SD were flagged and retained as a separate set of samples. Samples within 2 SD of the mean were denoted “the core set”.

To obtain a final call set, we redid the combineGVCF and genotypeGVCF steps with only the core set of samples. After VQSR, the final set of SNVs and Indels were genotyped in the flagged set of samples, and these were added to form the total set of 2.628 exomes.

**References**

1. Li H, Durbin R. Fast and accurate short read alignment with Burrows–Wheeler transform Bioinformatics. 2009 Jul 15; 25(14): 1754–1760.

2. McKenna A, Hanna M, Banks E, Sivachenko A, Cibulskis K, Kernytsky A, et al. The Genome Analysis Toolkit: a MapReduce framework for analyzing next-generation DNA sequencing data. Genome Res. 2010 Sep;20(9):1297-303.

3. <http://broadinstitute.github.io/picard/>) PNPTU.
